# An Improved Modeling and Numerical Analysis Method for Tooth Surface Wear of Double-Arc Harmonic Gears

**DOI:** 10.3390/ma15248869

**Published:** 2022-12-12

**Authors:** Qian Zhao, Zuoxiang Xing, Jing Yuan, Zhijun Zhang, Jun Zhu, Huiming Jiang

**Affiliations:** 1 School of Mechanical Engineering, University of Shanghai for Science and Technology, Shanghai 200093, China; 2 Shanghai Radio Equipment Institute, Shanghai 201101, China

**Keywords:** harmonic gear, wear evaluation, mixed elastohydrodynamic lubrication (EHL), Archard model, meshing offset

## Abstract

Tooth surface wear is one of the most common failure modes of harmonic gears, especially in space drive mechanisms. Due to difficulty accurately modeling its wear failure model and the complex mechanism, its dynamic behavior and wear mechanism have not been deeply investigated, and study of the double-arc tooth profile wear model is relative lacking. Therefore, an improved wear modelling and analysis method that is more in line with actual conditions for double-arc harmonic gears is here proposed. Firstly, a tooth surface wear model under mixed elastohydrodynamic lubrication (EHL) was established based on the Archard formula, which combines the Reynolds equation and double-arc tooth profile equation, and considering the meshing offset caused by elastic deformation. Then, the wear analysis method combined with mixed EHL was derived, and numerical simulation analysis of the wear characteristics in lubrication state was carried out, including wear depth calculation and wear output comparison of different tooth profiles. Furthermore, the influence of main working parameters and design parameters on the wear quantity was analyzed. The results show that wear depth for mixed EHL is significantly less than at dry contact. The double-arc tooth profile can withstand more wear cycles than the involute tooth profile, and the input torque and the number of cycles significantly affect the amount of tooth wear. This study further reveals the tooth wear mechanism for harmonic gears, and provides a theoretical basis for the structural optimization design, wear reduction, and life prolonging of harmonic gears.

## 1. Introduction

Harmonic drive gears are widely used in aerospace and industrial robotics due to the advantages awarded by their small size and light mass. Its failure modes include not only fatigue fracture of the flexspline but also tooth surface wear of gear teeth [1]. In particular, tooth surface wear leads to changes in the distribution of morphology and load, which will not only reduce the efficiency and stability of the harmonic transmission system, but also cause the generation of vibration and noise, and reduce the service life. Therefore, it is of great engineering significance to study the tooth surface wear mechanism of harmonic gear transmission and reveal the influence law of working condition parameters and design parameters on tooth surface wear, which is of great significance for vibration suppression, noise reduction, and life extension of the harmonic transmission system.

At present, the fatigue fracture failure of harmonic drive gear in the flexspline has been studied in depth. However, research on the numerical method of harmonic gear wear is very lacking. This is due to the particularity of the harmonic drive gears, which are always subjected to periodic elastic deformation, and the fact that the flexspline teeth are approximately elliptical when working. Therefore, the motion of the flexspline in relation to the circular spline becomes extremely complicated. The existing research mainly focuses on experimental research based on lubrication direction. Schafer et al. [2] conducted a thermal vacuum test on harmonic gears to evaluate the action law of various parameters, including load torque and operating speed, and analyzed the relationship between lubrication and wear. Gill et al. [3] experimentally studied the wear state of solid lubrication, oil lubrication, and grease lubrication under specific operating conditions using different lubrication methods in a thermal vacuum environment. Hans et al. [4] carried out the life test on harmonic drive gears and studied the influence of lubricating materials on tooth surface wear when using the grease lubrication method, and gold plating between the flex and circular spline teeth. Maniwa et al. [5,6] conducted a life experiment using MAC hybrid lubrication to examine the wear state of each contact pair under atmospheric and vacuum conditions. Li [7,8] conducted a 5000-h vacuum life test on drive performance to analyze the effects of rotational speed, load, and temperature on tooth surface wear, and to investigate the wear mechanism of a space-lubricated harmonic drive. However, the experimental method is costly, time-consuming, and lacks universality. Raviola et al. [9] experimentally studied the effect of wear on different harmonic gears’ transmission performance and torsional stiffness. They analyzed the relationship between hysteresis loss and torque based on hysteresis curves.

Over recent years, scholars based locally and abroad have conducted a lot of research on gear wear problems, but the progress of the development of numerical methods for tooth wear is limited by the fact that both the wear mechanism and lubrication mechanism of harmonic gears are more complex. At present, there is no completely universal theoretical formula, and most scholars still use the Archard wear formula for research. Hugnell et al. [10] used a single point observation method to model the wear of cams and followers and contrasted changes in contact pressure and surface morphology, which were also compared with wear to derive the wear pattern. E.N. Ivsshov et al. [11] put forward a wear calculation model for harmonic drives and carried out numerical simulation, but the model had certain errors due to the use of many assumptions. Bo [12] established a tooth wear model for involute harmonic gears, proposed the correction of tooth contact pressure using the meshing angle, and studied the influence of load torque on the wear of the flexspline. Jia [13] established the force equation of gear teeth to reduce the wear of the flexspline and analyzed the variation of friction force and friction factor with speed under lubrication conditions. Andersson et al. [14] proposed the famous ‘single-point observation method,’ based on the Archard wear equation, and calculated the wear depth at specific points on spur gears. In order to enhance the computation of helical gear wear, Flodin et al. [15,16] modified the equation presented by Andersson based on the simplified Winkler model. Karpat et al. [17] investigated the effect of different tip relief modifications and pressure angles on the wear of asymmetric gears. Mert et al. [18] applied the Archard formula to internal gears and conducted a theoretical study on the wear of the system. In addition, numerous academics have extended the models mentioned above to other gears, such as planetary gears [19,20], hypoid gears [21], irregular tooth forms [22], etc. Zhang et al. [23] established an involute gear tooth wear model under quasi-static operating conditions and analyzed the effects of gear meshing deviation, load, and the number of cycles on the wear depth of spur gears. Zhou et al. [24,25] proposed a calculation method for adhesive wear of helical gears, simulated the wear characteristics, and analyzed the influence law of different parameters on the amount of wear. Chen et al. [22,26] investigated the relationship between gear deviation and meshing stiffness and proposed a general analytical model. An improved analysis method for gear pairs with tip clearance is proposed by Ma [27,28]. However, there are too few wear numerical simulation studies on harmonic drive gears, mainly focusing on other gear types.

Due to the Archard model’s derivation from the dry friction state, the majority of Archard models used in the literature have significant restrictions. The harmonic drive analyzed in this study is used in a space-pointing mechanism, which often operates at low speed with a light load. The contacting subsets using lubrication within the reducer are frequently in a mixed or boundary lubrication state. Therefore, the analysis of the wear mechanism cannot be separated from the lubrication analysis considering the micro-convex body contact on the rough surface, which should be developed based on the adhesion wear model established by Archard in the dry contact condition. Li et al. [29] conducted accelerated life experiments on harmonic drives in a lubricated condition and showed that the adhesive wear caused by the contact of the micro-convex body is the main cause of the life. Luo et al. [30] proposed a new gear dynamics model to investigate the effect of EHL film during gear failure. Xiao et al. [31] studied the wear mechanism of herringbone gears under mixed lubrication and explored the effects of torque and speed on the minimum oil film thickness. Wang et al. [32] established a wear model for helical gears using mixed EHL and investigated the relationship between sliding distance, pressure, and wear depth. However, there are relatively few studies on various gears using mixed EHL, and even fewer studies on wear related to harmonic drive gears using mixed EHL.

In summary, much progress has been made in the numerical simulation of the wear of harmonic drives. However, in the above numerical simulation, the wear depth of gear teeth using lubrication is not investigated, and the coupling effect of various parameters on the wear amount is not fully discussed. In addition, the influence of meshing deviation and load on wear is rarely considered in other gear systems, such as straight teeth, helical teeth, internal meshing teeth, and external meshing teeth. In view of this, this paper proposes a wear analysis method for harmonic drive gears. In Section 3, a double-arc harmonic drive gear tooth surface wear model using mixed EHL is established based on the Archard wear formula. A numerical simulation analysis of the wear characteristics is carried out in Section 4. Section 5 analyzes the influence of the main working parameters (number of wear cycles, load torque) and design parameters (modulus, tooth width) on the wear quantity. Conclusions are drawn in Section 6.

## 2. Double-Arc Tooth Profile Design

**Figure 1:**
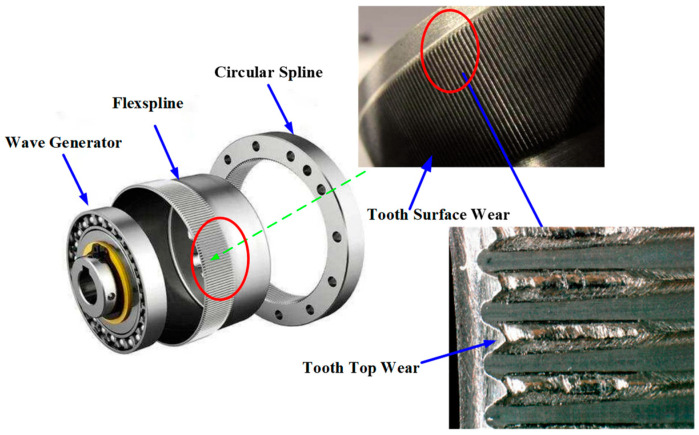
Flexspline tooth surface wear of a harmonic drive gear.

Harmonic drive gears mainly consist of a flexspline, circular spline, and wave generator. The main form of failure is tooth surface wear of the flexspline, and a worn gear is shown in Figure 1. By observing the local wear diagram of the gear, we can see that when serious wear occurs on the flexspline [9], it is mainly concentrated on the top part of the tooth, and the wear at the root is very minor. A section of the bottom of the double-arc tooth profile is not involved in the engagement; thus, there is no amount of wear. It is necessary to establish the tooth profile first, as the wear of gear teeth makes the tooth profile change constantly.

**Figure 2:**
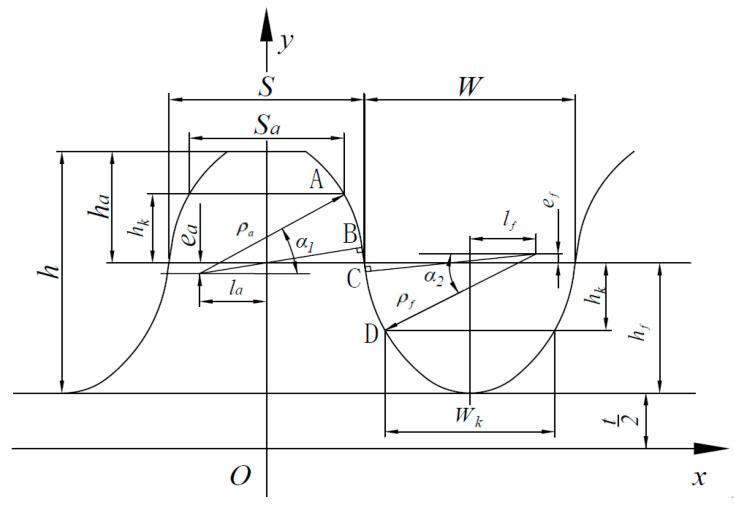
Double-arc tooth profile of flexspline.

The flexspline double-arc tooth profile is composed of two eccentric circles and a tangent line. The tooth profile structure and the relationship between parameters are shown in Figure 2. AB and CD are two circular arcs, and BC is the common tangent of the two arcs. The parameter equations for each part of flexspline are as follows:

Convex arc segment AB—see Equation (1)

(1) $$\begin{array}{c} \{\begin{array}{c} x_{1}=\rho_{a}\cos\alpha_{1}+l_{a}\\ y_{1}=\rho_{a}\sin\alpha_{1}-e_{a}+h_{f}+t \end{array}\left(\theta_{B}\leq \alpha_{1}\leq \theta_{A}\right) \end{array}$$

The tangent line segment BC—see Equation (2)

(2) $$\begin{array}{c} \{\begin{array}{c} x_{2}=x\\ y_{2}=kx+b_{1} \end{array}\;\left(\rho_{a}\cos\theta_{B}+l_{a}\leq x\leq \frac{S+W}{2}+l_{f}-\rho_{f}\cos\theta_{C}\right) \end{array}$$

Concave arc segment CD—see Equation (3)

(3) $$\begin{array}{c} \{\begin{array}{c} x_{3}=\frac{S+W}{2}+l_{f}-\rho_{f}\cos\alpha_{2}\\ y_{3}=t+h_{f}+e_{f}-\rho_{f}\sin\alpha_{2} \end{array}\;\left(\theta_{C}\leq \alpha_{2}\leq \theta_{D}\right) \end{array}$$

From the relationship between the line segments, it is determined that

(4) $$\theta_{C}=\pi +\theta_{B}\;k=\tan\left(\frac{\pi }{2}+\theta_{B}\right)=-\cot\theta_{B}\;k\left(\rho_{a}\cos\theta_{B}+l_{a}\right)+b=\rho_{a}\sin\theta_{B}-e_{a}+h_{f}+t\;k\left(\frac{S+W}{2}+l_{f}-\rho_{f}\cos\theta_{C}\right)+b=t+h_{f}+e_{f}-\rho_{f}\sin\theta_{C}$$

where $\rho_{a}$ and $\rho_{f}$ are the radius of convex and concave tooth arcs, respectively; $l_{a}$ and $l_{f}$ are the offset of convex and concave tooth centers, respectively; and $e_{a}$ and $e_{f}$ are the offset of convex and concave tooth centers, respectively. k is the slope of the common tangent line, b is the intercept, S is the thickness of convex pitch wheel teeth, W is the width of concave pitch tooth groove, $h_{f}$ is the root height, and t is the distance from the root to the transverse coordinate. The specific parameter size is derived from the empirical formula.

**Figure 3:**
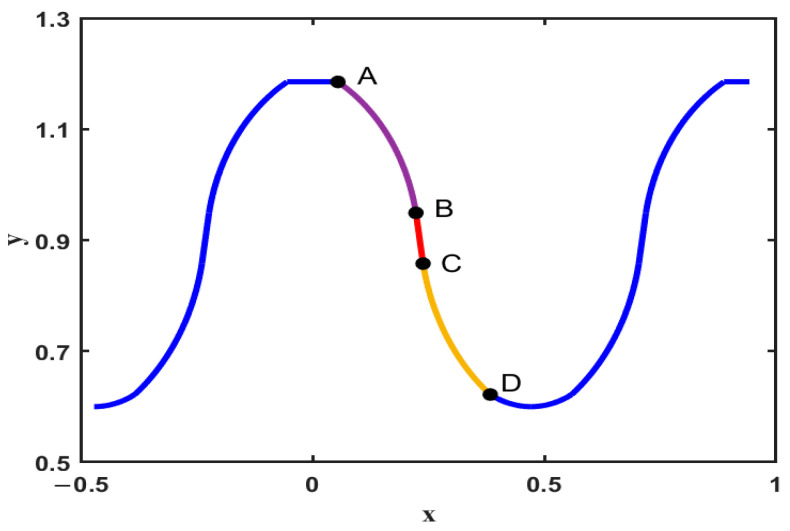
Tooth profile of the fitted flexspline.

Using the design parameters and the particle swarm algorithm, the tooth profile of the flexspline drawn by MATLAB is shown in Figure 3.

## 3. Wear Model in Harmonic Drive Gear

### 3.1. Wear Formula and Calculation Process

Sliding and rolling occur between the contact tooth surfaces of the flexspline and circular spline during the meshing process of harmonic drive gears, and both the contact load and sliding distance vary over time. In this study, the tooth surface wear model of harmonic drive gears was developed by enhancing the adhesive wear calculation formula proposed by Archard [33,34]. The classical Archard wear formula can be expressed as

(5) $$\begin{array}{c} \frac{V}{S}=K\frac{W}{H} \end{array}$$

where V is the wear volume, S is the sliding distance, K is the wear coefficient, W is the positive pressure on the wear surface, and H is the material hardness.

It is more meaningful to study the depth of wear than the wear volume in studying the wear mechanism. The wear volume and positive surface pressure can be calculated as

(6) $$\begin{array}{c} V=A\cdot h \end{array}$$

(7) $$\begin{array}{c} W=p\cdot A \end{array}$$

where A is the contact area, h is the wear depth, and p is the contact stress. Incorporating Equations (6) and (7) into Equation (5), the wear depth can be described as

(8) $$\begin{array}{c} h=\frac{K}{H}\cdot p\cdot s \end{array}$$

Currently, Archard’s formula is still the most widely used in the wear study. However, the wear depth calculated by the traditional formula has a significant error. This is due to the fact that Archard formula is based on dry contact and does not account for the actual state of gear operation. Fluid lubrication is classified into four states according to the Stribeck curve in fluid lubrication: hydrodynamic lubrication, elastic fluid dynamic pressure lubrication, mixed lubrication, and border lubrication. Li et al. [35] studied the failure mechanism of space-lubricated harmonic driver and concluded that the harmonic gear teeth mostly operate in a mixed EHL state. The contact load in this state is shared by the lubricant film and the micro-convex body. To evaluate the wear rate when using mixed EHL, Masjedi [36] extended the Archard formulation derived from dry conditions to the lubrication state and proposed an EHL formulation for dealing with rough surfaces by solving the modified Reynolds equation.

To obtain the wear equation of mixed EHL, the total load needs to be replaced by the partial load carried by the micro-convex body as the roughness loading ratio, $L_{a}$. Additionally, the lubricating molecules on the surface significantly reduce the wear rate as the fractional film defect, ψ. The two parameters can be expressed as follows:

(9) $$\begin{array}{c} L_{a}=0.005W^{-0.408}U^{-0.088}G^{0.103}\left[ln\left(1+4470\sigma^{6.015}V^{1.168}W^{0.485}U^{-3.741}G^{-2.898}\right)\right] \end{array}$$

(10) $$\psi =1-exp\left\{-\left[\frac{\alpha_{x}}{u_{s}t_{0}}exp\left(-\frac{E_{a}}{R_{g}T_{s}}\right)\right]\right\}$$

The wear equation for mixed EHL can be expressed as [37]

(11) $$\begin{array}{c} h=\frac{K}{H}\cdot \psi \cdot \left(\frac{L_{a}}{100}\right)\cdot p\cdot s \end{array}$$

**Figure 4:**
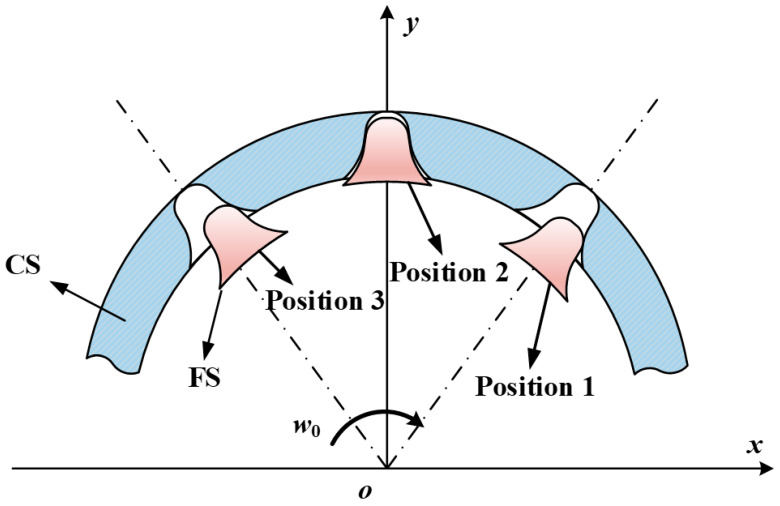
The engagement process.

The wear depth of each position on the tooth profile is different. This is because the contact pressure and sliding distance of each point on the gear teeth are different. Therefore, when performing wear analysis, the Archard wear model needs to be discretized based on the gear meshing process, as given in Figure 4. While the wear coefficient and material hardness of each contact point remain unchanged, the wear equation of each contact point *k* after dispersion is

(12) $$\begin{array}{c} h_{k}=\frac{K}{H}\cdot \left(\frac{L_{a}}{100}\right)\overset{n}{\underset{i=k}{\sum }}\psi_{i}p_{i}s_{i} \end{array}$$

where i is the number of wear cycles at each discrete point.

**Figure 5:**
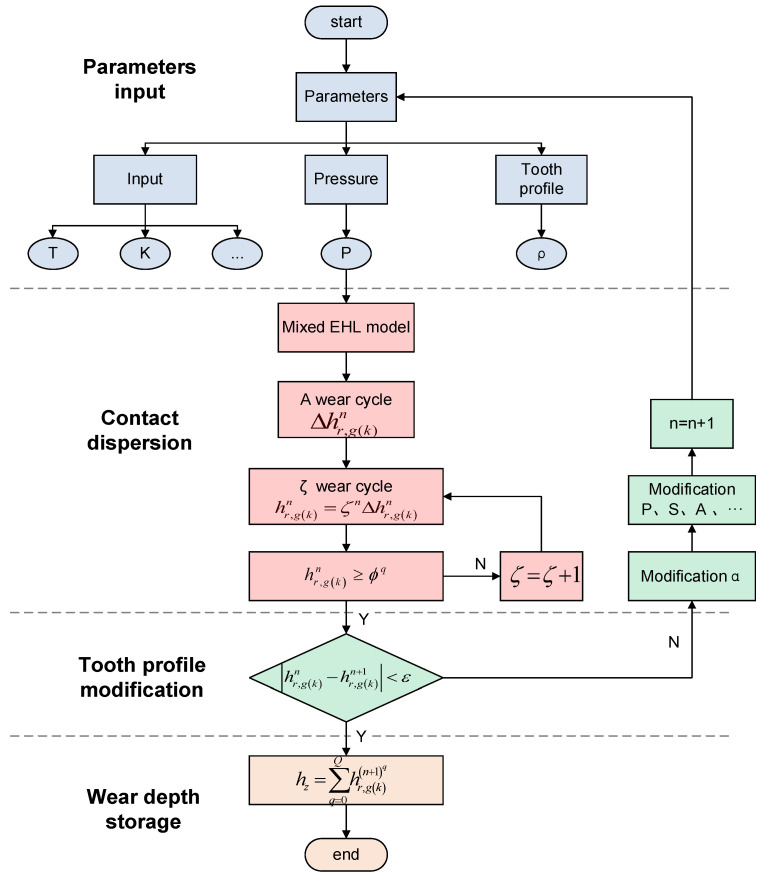
Wear numerical simulation process.

Based on the above equation, the wear depth after material removal is simulated by numerical method, and the wear numerical simulation flow is illustrated in Figure 5.

First, the original parameters, including torque, pressure, and tooth profile, need to be input into the program. Wear is calculated based on the established mixed EHL model. To determine the wear depth at any position along the contact line, it is necessary to discretize the functioning tooth profile. A point on the tooth surface achieves threshold $\phi^{q}$ after wear cycles during the process of gear wear. At this time, the slope angle of each discrete point continuously changes, as the tooth profile changes during the wear process. The slope angle obtained after each wear correction is used to correct other variables in the wear equation, such as pressure (p), sliding distance (s), contact area (A), sliding speed ($u_{s}$), etc. Using this method, the wear-related variables are prevented from being constant or changing infrequently, and the time-varying nature and precision of the solution are significantly enhanced. Once the difference between *n* correction cycles and *n* + 1 correction cycles is less than the preset accuracy threshold ε, in other words $\left|h_{r,g(k)}^{n}-h_{r,g(k)}^{n+1}\right|<\varepsilon$, then the cycle ends.

### 3.2. Contact Pressure

**Figure 6:**
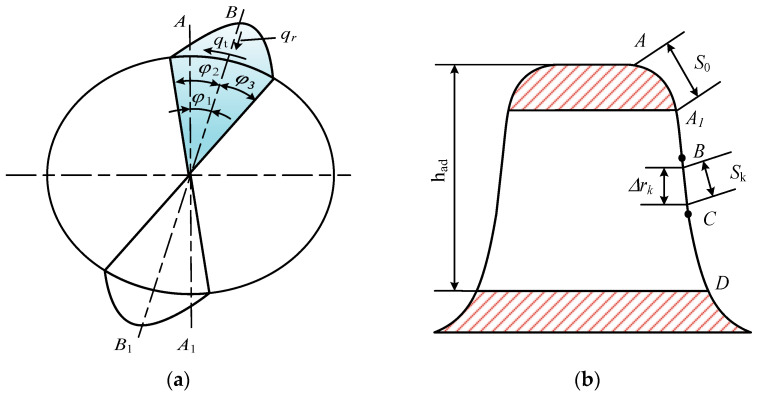
Gear teeth load distribution: (**a**) in its entirety, and (**b**) for a single tooth.

The load distribution on the teeth of flexspline and circular spline during differential tooth motion of harmonic drive gear is extremely complicated. It is dependent not only on the periodic elastic deformation caused by the bracing of the wave generator, but also on the input torque, tooth stiffness, assembly, and manufacturing. Therefore, it is difficult to express the load situation on harmonic gear teeth using equations. Kayabasi et al. [38] experimentally obtained the approximate relationship of load distribution as shown in Figure 6, where $\varphi_{2}$ and $\varphi_{3}$ are the the engagement region angles, and $\varphi_{1}$ is the angle between the centers of the engagement regions BB_1_ and AA_1_ at the wave generator’s long axis support. According to the ring theory and the meshing principle of harmonic gear teeth, the two angles $\varphi_{2}$ and $\varphi_{3}$ are considered to be approximately equal. The unit width load on the tooth surface in each direction in the $\varphi_{2}$ region can be expressed as

(13) $$\{\begin{array}{c} q_{t}=q_{tmax}\cos\left[\pi \left(\varphi -\varphi_{1}\right)/2\varphi_{2}\right]\\ q_{r}=q_{t}\tan\alpha^{\prime } \end{array}$$

The unit width load of tooth surface in each direction in the $\varphi_{3}$ region can be expressed as

(14) $$\{\begin{array}{c} q_{t}=q_{tmax}\cos\left[\pi \left(\varphi -\varphi_{1}\right)/2\varphi_{3}\right]\\ q_{r}=q_{t}\tan\alpha^{\prime } \end{array}$$

where $q_{t}$ is the tangential load applied to the tooth per unit width, $q_{r}$ is the radial load applied to the tooth per unit width, and $\alpha^{\prime }$ is the pressure angle at the contact point.

As the input of harmonic gear teeth, the flexspline is subjected to a certain moment, and the relationship between moment *T* and tooth load can be described as follows:

(15) $$T=4\int_{\varphi_{1}}^{\varphi_{1}+\varphi_{2}}b{\left(d_{g}/2\right)}^{2}q_{tmax}\cos\left[\pi \left(\varphi -\varphi_{1}\right)/\left(2\varphi_{2}\right)\right]d\varphi$$

Integrating the equation yields the following:

(16) $$q_{tmax}=\pi T/\left(2\varphi_{2}d_{d}{}^{2}b\right)$$

where b is the width of the tooth ring of the flexspline, $d_{g}$ is the index circle’s diameter, and $d_{d}$ is the tooth contact point’s equivalent diameter. The relationship between the two diameters can be defined as follows:

(17) $$\begin{array}{c} d_{d}=d_{g}-h_{ad} \end{array}$$

where $h_{ad}$ is the height of the teeth involved in the engagement.

To solve the stresses occurring on the harmonic gear teeth, it is necessary to integrate the meshing area angles $\varphi_{2}$ and $\varphi_{3}$ by segment, with the length of each segment being equal to one tooth pitch. Assuming that the angles corresponding to the ends of each segment length are $\varphi_{k}$ and $\varphi_{k+1}$, the tangential stress $f_{t}$ on this single tooth can be obtained as follows:

(18) $$f_{t}=\int_{\varphi_{k}}^{\varphi_{k+1}}b\left(d_{g}/2\right)q_{tmax}\cos\left[\pi \left(\varphi -\varphi_{1}\right)/\left(2\varphi_{2}\right)\right]d\varphi$$

Discrete for the contact points of the working tooth segment, the calculation for the tangential stress at each contact point will be replaced by the following equation:

(19) $$f_{tk}=b_{R}\left(d_{ag}/2\right)q_{tmax}\cos\left\{\pi \left[\varphi_{1}+\varphi_{2}-\left(k-0.5\right)\Delta \varphi -\varphi_{1}\right]\right\}\Delta \varphi /\left(2\varphi_{2}\right)$$

The force applied to the tooth is obtained as follows:

(20) $$f_{k}=f_{tk}/\sin\alpha_{k}$$

The contact pressure can be expressed as

(21) $$p_{k}=f_{k}/A_{k}$$

### 3.3. Sliding Distance

This paper focuses on the double-arc tooth profile established in the preceding section. The eccentric circle radius and the operating angle can express the sliding distance between the two arcs and the tangent line. The sliding distance of each segment can be calculated as follows.

Convex arc segment AB—see Equation (22)

(22) $$\begin{array}{c} s_{k}=\frac{\rho_{a}\pi \left(\theta_{B}-\theta_{k}\right)}{180}\;\;\;\;\left(\theta_{B}\leq \theta_{k}\leq \theta_{A}\right) \end{array}$$

The tangent line segment BC—see Equation (23)

(23) $$\begin{array}{c} s_{k}=s_{AB}+\sqrt{{\left(x_{k}-x_{B}\right)}^{2}+{\left(y_{k}-y_{B}\right)}^{2}} \end{array}$$

The parameter relationship of $y_{k}$ is given by Equation (2).

Concave arc segment CD—see Equation (24)

(24) $$\begin{array}{c} s_{k}=s_{AB}+s_{BC}+\frac{\rho_{f}\pi \left(\theta_{D}-\theta_{k}\right)}{180}\;\;\;\;\;\left(\theta_{D}\leq \theta_{k}\leq \theta_{C}\right) \end{array}$$

### 3.4. Contact Area

The contact area is generally the product of the sliding distance between the contact point and the tooth width. When the flexspline and the circular spline first begin to mesh, the contact area can be expressed as

(25) $$\begin{array}{c} A_{0}=s_{0}b \end{array}$$

Then the contact area of the *k*-th point can be obtained with using the equation

(26) $$\begin{array}{c} A_{k}=A_{0}+b\overset{k-1}{\underset{i-1}{\sum }}si \end{array}$$

**Figure 7:**
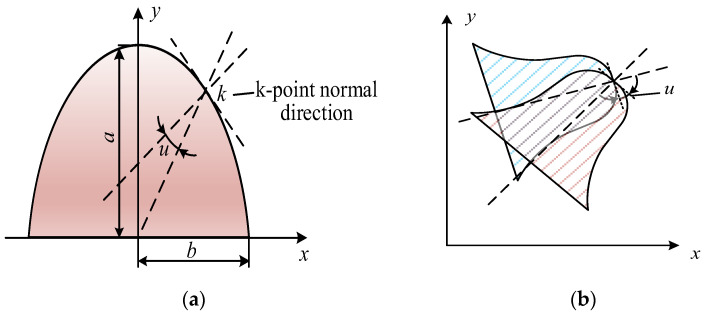
Gear tooth deviation: (**a**) in its entirety; (**b**) for a single tooth.

The above expression for the contact area of each discrete point applies to general gears. The flexspline engages will deflect, resulting in a change in the contact area. This is because it is propped up by the wave generator, which will produce periodic elastic deformation into an ellipse during operation. The deflection angle of the teeth of the flexspline is presented in Figure 7, and the deflection (*u*) can be used in this simulation as follows [12]:

(27) $$\begin{array}{c} u=\left|\arctan\left(-\frac{a}{b}\tan\varphi \right)\right|-\left|\varphi \right| \end{array}$$

After calculating the deflection of the gear’s teeth, the contact area can be expressed as

(28) $$\begin{array}{c} A_{k}=A_{0}+b\overset{k-1}{\underset{i=1}{\sum }}s_{i}\pm \frac{bus_{ag}}{2} \end{array}$$

where ‘+’ is used to denote engaging in, ‘−’ is used to denote engaging out, and $s_{ag}$ is the tooth top thickness.

### 3.5. Wear Coefficient (K)

In the listed mixed lubrication wear equation, the wear coefficient (*K*) is the critical parameter affecting the wear depth. However, the wear coefficient varies significantly under different working conditions and is more difficult to obtain, as it is influenced by surface roughness, operating environment, material properties, lubrication conditions, etc. Janakiraman [39] obtained the regression equation for the wear coefficient by performing statistical analysis for different loads, speeds, lubrication conditions, and surface roughness, with the specific expression:

(29) $$\begin{array}{c} K=\frac{3.981\times 10^{29}}{E^{*}}L^{1.219}G^{-7.377}S^{1.589} \end{array}$$

where $E^{*}$, *L*, *S*, and *G* are the moduli of elasticity, gauge load, gauge-surface roughness, and gauge-viscosity coefficients, respectively. Each coefficient is calculated as follows:

(30) $$\begin{array}{c} L=\frac{W^{\prime }}{E^{\prime }R^{\prime }}\;G=\alpha E^{\prime }\;S=\frac{R_{\alpha }{}^{c}}{R^{\prime }} \end{array}$$

where $W^{\prime }$, α, and $R^{\prime }$ are the unit load, pressure–viscosity coefficient (only affected by temperature, not pressure), and equivalent radius, respectively. $R_{\alpha }{}^{c}$ is the composite roughness, and its formula is as follows:

(31) $$\begin{array}{c} R_{\alpha }{}^{c}=\sqrt{R_{\alpha_{1}}+R_{\alpha_{2}}} \end{array}$$

where $R_{\alpha_{1}}$ and $R_{\alpha_{2}}$ are the surface roughness of the two gears. The regression equation demonstrates that the wear coefficient is positively related to both load and surface roughness, but negatively related to the viscosity coefficient. The effect of the viscosity coefficient was the most significant, while the effect of load was the least significant. The harmonic drive studied in this paper works in a space pointing mechanism, so the coefficients should be selected in accordance with the ambient space temperature.

### 3.6. Calculation of Wear Depth

Wear is essentially a process of material removal. The amount of change in the tooth profile at each point is the amount of wear in numerical simulation. After the wear cycle, the shape of the tooth profile changes, so whether it is an involute tooth profile or a double-arc tooth profile, the slope angle ($\alpha_{k}$) of each discrete point varies continuously with the wear cycle. The current wear cycle slope angle needs to be calculated, and then the contact pressure, contact area, and other parameters listed above are corrected using the current slope angle, in order to calculate the wear volume in the next cycle. The accuracy of the output quantity is ensured by continuously cycling the above steps.

**Figure 8:**
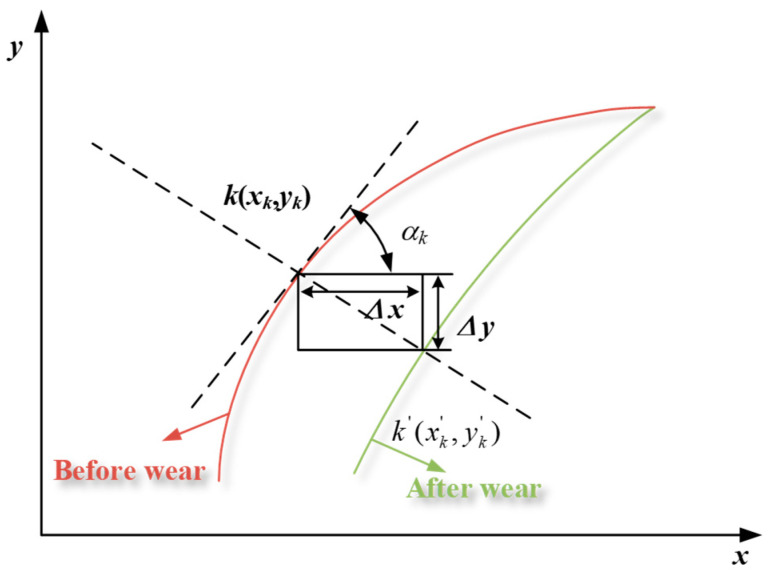
Correction of tooth profile.

The changes in tooth profile and slope angle before and after the wear of harmonic gear is displayed in Figure 8. Before wear, the coordinates of a meshing point (*k*) are $\left(x_{k},y_{k}\right)$, and after a period of wear, the coordinates are $\left(x_{k}{}^{\prime },y_{k}{}^{\prime }\right)$. The profile change is shown below:

(32) $$\{\begin{array}{c} x_{k}{}^{\prime }=x_{k}+h_{k}\sin\alpha_{k}\\ y_{k}{}^{\prime }=y_{k}+h_{k}\cos\alpha_{k} \end{array}$$

where the slope angle $\alpha_{k}$ is calculated as follows:

(33) $$\alpha_{k}=arc\tan\frac{y_{k+1}-y_{k}}{x_{k+1}-x_{k}}$$

## 4. Wear Simulation

### 4.1. Parameters of the Calculation Case

The basic parameters of the flexspline and circular spline required for the simulation are listed in Table 1. The parameters in the table can be obtained from the gear calculation formula and the material properties themselves. This work compensates the coupling relationship between parameters and wear depth. The contact pressure and sliding distance can be calculated through the above simulation process and the built wear model. The following analyses all include deflections due to elastic deformation. The double-arc and the conventional involute tooth profile model are also compared and analyzed to study the tooth surface wear mechanism of harmonic gears in depth.

### 4.2. Contact Pressure

**Figure 9:**
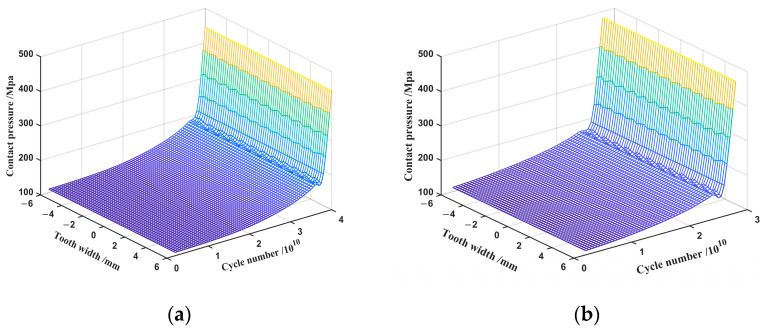
Contact pressure: (**a**) double-arc tooth profile; (**b**) involute tooth profile.

This analysis is based on the calculation parameters and wears cycle flow described above. The wear simulation analysis was conducted under the following operating conditions: an input torque of 16 Nm, an input speed of 1200 rpm, and a swing angle of ±36°. In order to simulate the realistic conditions of harmonic tooth wear, the amount of wear in and out of engagement is defined as equal, which is due to the fact that wear exists on both the left and right sides of the gear teeth. The variation in the average contact pressure with tooth width and number of wear cycles, as obtained by contact analysis, is displayed in Figure 9. It can be seen that both double-arc and involute tooth profiles have a more uniform contact pressure distribution along the tooth width direction in ideal conditions. The average contact pressure is directly proportional to the cycle number. With wear cycles, the average contact pressure on the tooth surface gradually increases. After reaching a certain number of cycles, the contact pressure rises rapidly, at which time the harmonic gear tooth changes from the stable wear stage to the severe wear stage, and the gear tooth is damaged. As seen in Figure 9a, the average contact pressure of the double-arc tooth profile increased dramatically after 4 × 10^10^ cycles, reaching 444.66 MPa. As presented in Figure 9b, the average contact pressure of the involute tooth profile changed in the same way after 2.8 × 10^10^ cycles, with the contact pressure value increasing to 481.67 MPa. The double-arc tooth profile is able to take more wear cycles under the same operating conditions, as is made clear when comparing the two tooth profiles. The contact pressure of the double-arc tooth profile is less than that of the involute tooth profile during the whole operation.

### 4.3. Sliding Distance

**Figure 10:**
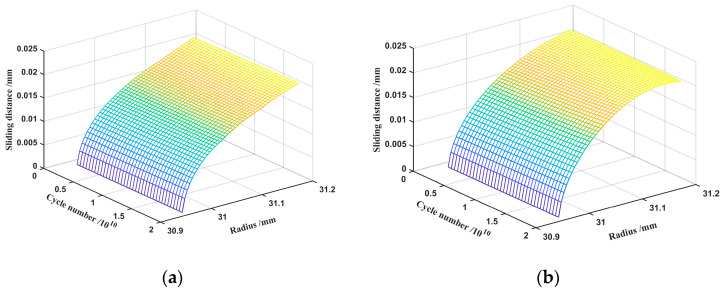
Sliding distance: (**a**) double-arc tooth profile; (**b**) involute tooth profile.

In the listed wear equation for mixed EHL, the tooth surface sliding distance, in addition to the contact pressure, is a critical element impacting the wear depth, which is proportional to the quantity of wear. Figure 10 shows the maximum sliding distance at the top of the flexspline teeth, which decreases as it approaches the root of the teeth. As wear continues, the sliding distance of each contact point on the working tooth profile has the tendency to fluctuate and increase overall. Figure 10b shows that the sliding distance at the top of the involute tooth profile is 0.022 mm after 2 × 10^10^ cycles. Figure 10a shows that the sliding distance of the top of the double-arc tooth profile is 0.021 mm after the same number of wear cycles. The sliding distance of the involute tooth profile is slightly greater than that of the double-arc tooth profile under the same working conditions. Overall, the effect of tooth profile on the sliding distance is almost negligible.

### 4.4. Wear Depth

**Figure 11:**
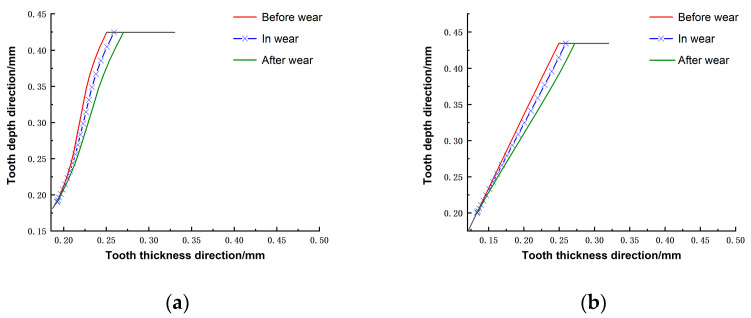
Wear tooth profile change: (**a**) double-arc; (**b**) involute.

Figure 11 shows the change in tooth profile after wear. The red curve indicates the tooth profile before wear, the blue curve indicates the tooth profile after 2.56 × 10^9^ wear cycles, the green line represents the tooth profile after 6.28 × 10^9^ wear cycles, and the black curve indicates the top of the flexspline and the part not affected by wear. It can be seen that the tooth surface wear of the harmonic gear is proportional to the number of wear cycles: the more cycles, the greater the wear depth. Whether it is a double-arc tooth profile or an involute tooth profile, the wear is the greatest at the top of the tooth, and the amount of wear gradually decreases from the top to the root of the tooth. The distribution of wear obtained from the simulation is consistent with the actual wear (Figure 1). This trend also corresponds exactly to the *x*-axis of Figure 10. This is due to the fact that the contact pressure (p) and sliding distance (s) at the tooth’s root is the greatest; therefore, the wear depth is the greatest here. There is a section at the root of the tooth that suffers no wear because it is not involved in the engagement.

**Figure 12:**
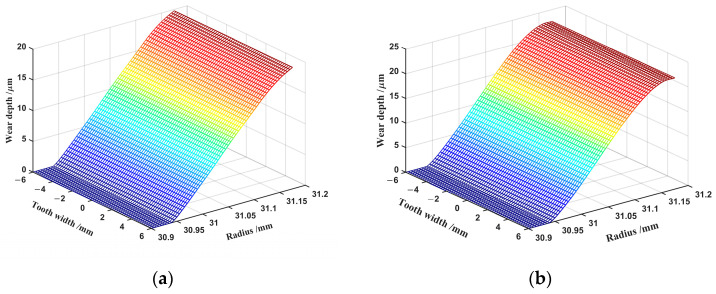
Wear depth: (**a**) double-arc tooth profile; (**b**) involute tooth profile.

In order to visualize the distribution of wear depth, Figure 12 depicts the wear depth along the tooth width and the radius of the flexspline under ideal conditions. The wear is uniformly distributed along the tooth width axis, with the same trend as that of Figure 9 along the *y*-axis. The output data from the numerical simulation show that the locations on the harmonic gear where the contact pressure is high have significant wear depths, and the locations where the contact pressure is low have slight wear depths. Equation (12) also shows that the contact pressure (p) is proportional to the wear depth. The contact pressure and the wear depth are increased by comparing Figure 9 and Figure 11 with the number of wear cycles. It can be inferred from this that engagement leads to a constant generation of wear, resulting in a subsequent increase in contact pressure, which further increases wear depth and wear rate. The two are mutually dependent.

The wear depth of the involute tooth profile is greater for all parts of the working tooth segment than that of the double-arc tooth profile, as shown by comparing Figure 12a,b. Therefore, the double-arc tooth profile can withstand more wear cycles than the involute tooth profile, as it has more wear resistance. In the design of harmonic drives considering wear, the double-arc tooth profile should be preferred.

### 4.5. Wear Depth by Different Methods

**Figure 13:**
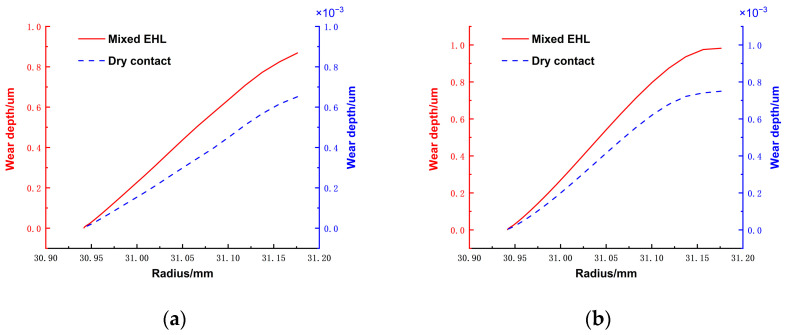
Wear depth along the radius for mixed EHL and dry contact: (**a**) double-arc tooth profile; (**b**) involute tooth profile.

Numerous studies in the literature [24] show that wear studies on various gear systems are mainly based on dry contact analysis. To compare the wear results obtained using this study’s method with previous methods [16], the graph shown in Figure 13 is plotted while ensuring that all other variables remain constant. The solid red line indicates mixed EHL, and the dashed blue line indicates dry contact. The variation curves show that the distribution of wear obtained by different wear analysis methods is similar, with the wear decreasing along the radius of the flexspline from the top of the tooth to its root. However, the wear depth in mixed EHL conditions is approximately three orders of magnitude less than in dry contact conditions. It can be inferred that a reasonable use of lubrication can effectively reduce wear. Since the harmonic drives operate at low speed with a light load, with mixed lubrication of the contacting parts [29], the output results on wear obtained in this research correspond more closely to the actual operating conditions of the harmonic drives.

## 5. Influence of Working Condition Parameters and Design Parameters on the Wear Depth

The analysis of the factors influencing the wear depth can provide reference significance for the selection of parameters, reductions in wear, and life extension.

### 5.1. Effect of Torque on Wear Depth

**Figure 14:**
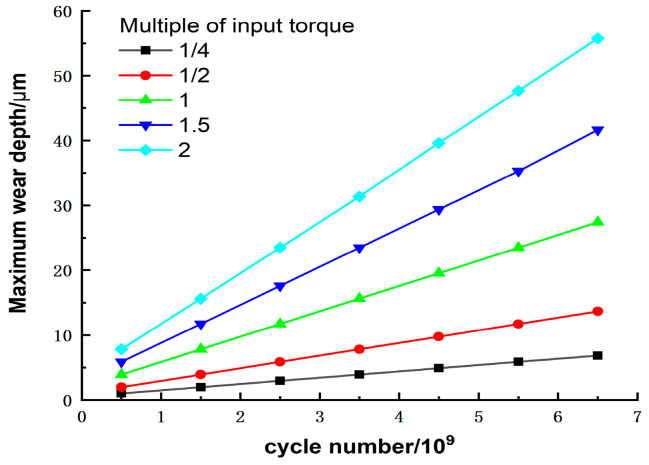
Effect of torque on maximum wear depth.

The variation of the maximum wear depth with the input torque and the number of wear cycles is shown in Figure 14. It can be seen that the input torque significantly affects the tooth surface wear depth. When the number of wear cycles remains constant, the higher the torque, the greater the maximum wear depth. At the same torque, the wear depth also increases with the number of wear cycles, which is the same conclusion as that obtained from Figure 11. As such, the wear depth is greatest at the maximum torque and cycle number. This is because the input torque is positively related to the load, and the contact pressure at each contact point of the tooth surface increases with increases in the load. The contact pressure and the wear depth are coupled with each other, so that an increase in either results in greater wear output.

Li et al. [29] conducted an experimental mixed lubrication analysis at various rotating speeds, and the load ratio of micro-convex body contact was equated with the wear state to achieve accelerated life. The numerical simulation laws obtained in this paper are consistent with these experimental results.

From the slope at each torque, it can be seen that the wear rate increases as the input torque increases. An increase in wear depth is more significant than an increase in torque; for example, in cycle number 6.52 × 10^9^, The maximum wear depth at two times the torque is 8.15 times that of 1 times the torque and 2.03 times that of 0.25 times the torque, respectively. This is because the larger the torque, the more pronounced the tooth profile change is over the same time period, and increases in the tooth surface force ($f_{k}$) and sliding distance (s) accelerate the wear rate simultaneously. Therefore, increasing the torque causes the harmonic gear teeth to wear more quickly. On the other hand, the wear rate also increases slowly with the wear cycle for the same reason as the torque in the case of a fixed torque. The increase in the wear rate is less pronounced during the stable wear phase, and the wear rate increases significantly when the gear tooth surface wear increases. In summary, the influence of torque needs to be considered in the design of harmonic drives to reduce wear and extend life.

### 5.2. Effect of Meshing Offset on Wear Depth

**Figure 15:**
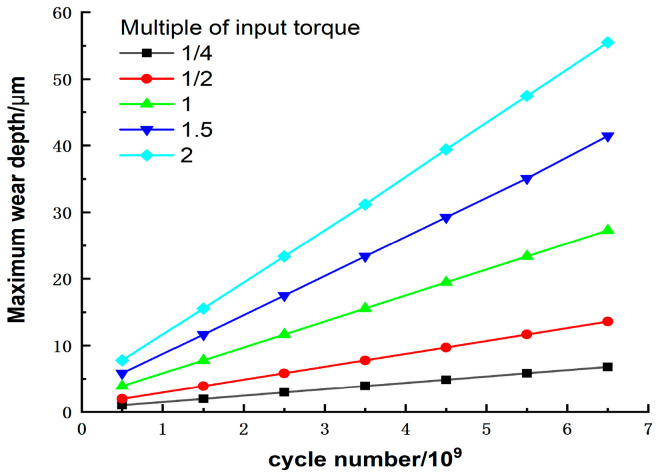
Effect of torque on maximum wear depth without offset.

In previous wear studies of double-arc harmonic drives, the engagement offsets were not considered, leading to errors in the model. To investigate this error, the effects of the input torque and the wear number on the wear depth, without considering the engagement offset, are illustrated in Figure 15. By comparing Figure 14 and Figure 15, it can be observed that the meshing offset does not affect the impact of the input torque or wear cycles on the wear depth, but only the value of the wear output. In general, the wear depth when considering the offset is slightly larger than when ignoring the offset. For example, the maximum wear depth of the double-arc tooth profile is 0.0557 mm with offset and 0.0554 mm without offset, after 6.52 × 10^9^ wear cycles at two times the torque. This is due to the fact that the meshing offset directly influences the contact area of the tooth surface, resulting in a change in the positive pressure, which in turn affects the wear depth.

**Figure 16:**
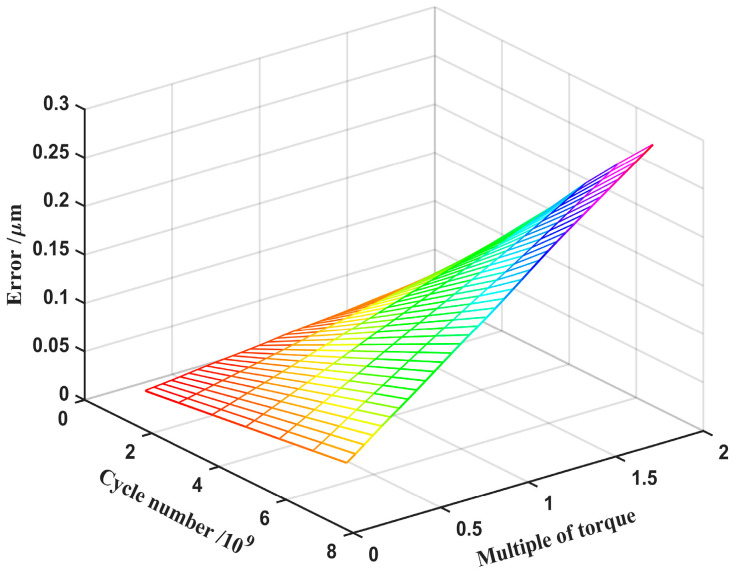
Error varies with input torque and number of cycles.

The value of the wear depth with and without the engagement offset is subtracted as the error amount for calculating the wear depth. The effect of the engagement offset on the wear depth is shown in Figure 16. It can be concluded that the errors are negligible when the number of cycles and the input torque are lower. However, the error amount increases more significantly with both the wear cycle and the increase in input torque, in which the influence of torque on the error amount is more evident than the number of cycles. Therefore, whether or not to consider the meshing offset can lead to differences in the wear depth. As the tooth surface wear becomes more severe, the amount of error generated is also more significant. The meshing offset due to elastic deformation must be accounted for in the study of the wear mechanism and life evaluation.

### 5.3. Effect of Modulus on Wear Depth

**Figure 17:**
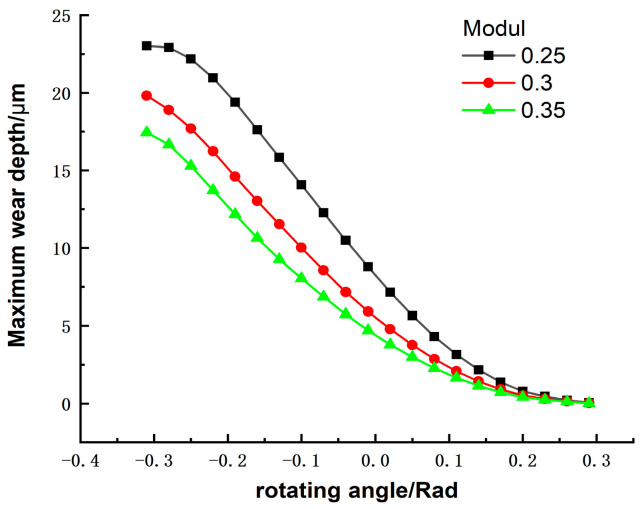
Effect of modulus on maximum wear depth.

The gear diameter is equal to the number of modules multiplied by the number of teeth. When the number of teeth is constant, the larger the modulus, the larger the diameter of the gear, and the greater the bending fatigue strength. However, the size of the harmonic drives cannot be too large in space, and a larger modulus will cause the overall model to exceed the design limits. Therefore, it is crucial to choose the appropriate modulus for harmonic drive gears. In this paper, we choose moduli of 0.25, 0.3, and 0.35 for the study of wear influence. The effect of modulus on wear depth is shown in Figure 17. In this study, we equated the radius to the rotating angle. When the modulus is different, the wear on the top of the tooth is still the largest and that on the root is the smallest. Therefore, the modulus does not change the distribution of the harmonic gear tooth wear. As the modulus increases, the tooth surface wear at each contact point of the working tooth profile tends to decrease. The pattern of wear depth change with modulus is also consistent with other studies’ findings [32]. The contact area of the engagement increases, and the contact pressure decreases at this time, leading to a reduction in wear depth. Therefore, the higher the modulus of the harmonic gear tooth, the more uniform the wear in the contact area of its profile.

### 5.4. Influence of Tooth Width on Wear Depth

**Figure 18:**
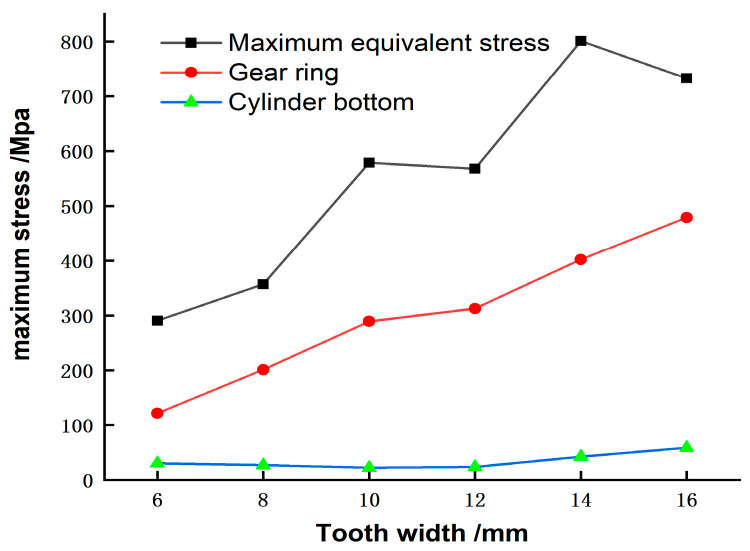
Stress transformation curve with tooth width.

In the process of designing and optimizing the harmonic drive, in order to reduce the fatigue fracture of the flexspline caused by cyclic elastic deformation, it is necessary to reduce its equivalent force. First, we must study the influence of parameters such as tooth width, barrel length, and wall thickness on the stress of the flexspline, and then perform multi-objective optimization. The effect of the resulting tooth width on the stress in the flexspline is shown in Figure 18. As the tooth width increases, the stress on all parts of the ring increases [40]. Therefore, selecting a narrower tooth width will effectively prevent tooth failure due to fatigue fracture.

**Figure 19:**
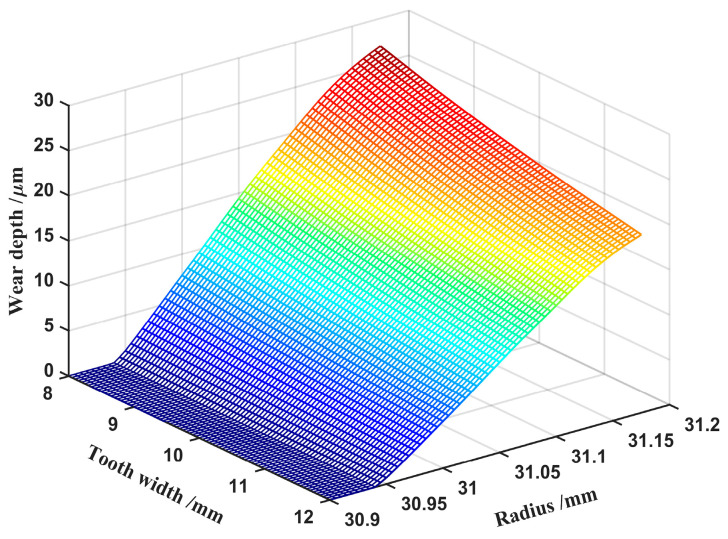
Effect of tooth width on wear depth.

Tooth wear is the main failure cause of harmonic drives, and most scholars only consider small, lightweight, and minimum stresses as optimization objectives, without considering how tooth width affects wear depth. Therefore, in order to reduce the failure of harmonic gear teeth and to prolong the life of the gear teeth, the influence of tooth width and wear depth is analyzed. Figure 19 shows that the wear depth increases with decreases in tooth width at all contact points of the harmonic gear teeth. The wear depth at the top of tooth has the highest growth rate, and the closer to the top of the tooth, the more dramatic the growth trend. The gradient of wear along the tooth height decreases as the tooth width increases from 8 mm to 12 mm. Therefore, the selection of smaller tooth widths will intensify the wear of the gear teeth, which conflicts with the goal of optimized design. It is thus clear that the selection of the tooth width of the harmonic drive teeth needs to be taken into account, both with regard to stress and wear depth, in order to prevent the failure rate of the harmonic drive from being too fast. Therefore, under the influence of the tooth wear depth, the tooth width should not be less than 10 to increase the service life of the harmonic drive. 

### 5.5. Influence of Hardness on Wear Depth

Hardness is a crucial variable in Archard’s wear equation, and while it is recognized that it is inversely related to the wear depth, the relationship between hardness and wear is more informative for the selection of specific values. 

**Figure 20:**
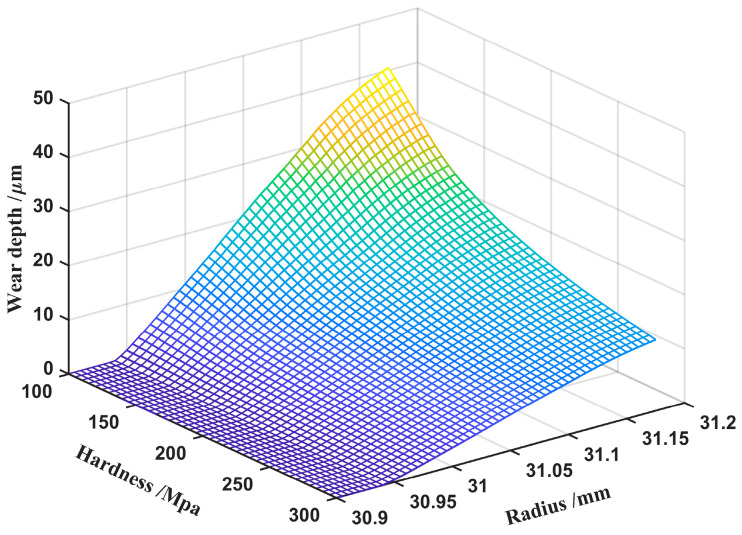
Effect of hardness on wear depth.

Figure 20 demonstrates that the wear depth of each contact point increases as the hardness decreases, whereas the effect of hardness on the wear depth of the tooth top is more pronounced. When the hardness is less than 200 MPa, the changing trend of the wear depth of each contact point is more drastic. Therefore, the selection of material hardness that is more than 200 MPa is appropriate.

## 6. Conclusions

In this research, an improved wear analysis method of harmonic gears that considers lubrication characteristics is proposed. A wear model of harmonic drive gear tooth surface using mixed EHL was established by considering the mesh offset caused by elastic deformation. The wear mechanism of the harmonic gear transmission system was studied through numerical simulation, the wear outputs of the double-arc tooth profile and the involute tooth profile were compared, and the effects of the working and design parameters on the wear depth were analyzed. The main conclusions are as follows.

1. Regardless of whether the established double-arc tooth profile or the conventional involute tooth profile is used, the contact pressure and wear depth of harmonic gears are more uniformly distributed along the tooth width direction under ideal conditions. The gradient of wear depth is larger along the working tooth profile direction, among which the wear depth in the top region is the largest. The wear amount gradually decreases towards the root region, and the wear depth in the root region of the working tooth segment is the smallest.
2. Different methods of wear analysis have no effect on the distribution of gear wear in harmonic drive gears. The mixed EHL wear model used in this paper corresponds more closely to the actual operating conditions of the harmonic drive, and the simulation results in a significantly smaller wear depth than that of dry contact. The use of proper lubrication can effectively reduce wear.
3. By comparing the double-arc profile and the involute tooth profile under the same working conditions, we concluded that the contact pressure, sliding distance, and wear volume of the involute tooth profile are larger than those of the double-arc tooth profile. The double-arc tooth profile can not only withstand more wear cycles, but also has stronger wear resistance.
4. The influences of both input torque and wear cycle number on the wear depth of harmonic gear teeth are significant. The wear depth of the tooth surface increases with an increase in input torque and cycle number, and the wear rate also increases monotonically with both variables. When the design goal is to extend the life of harmonic gears and reduce wear, the effects of service life and load must be considered.
5. The effects of modulus, tooth width, and hardness on the wear of gear tooth surface were analyzed. The results showed that increases in modulus, tooth width, and hardness can reduce the wear of tooth surfaces. Among them, tooth width and hardness have greater effects on the wear depth, and the selection of smaller tooth widths undermines the goal of optimized design. Using appropriate design parameters can improve the wear performance of tooth surfaces and prolong the service life of harmonic gear systems.
6. Research on modeling and analysis of traditional gear system have been relatively sufficient. Future research could focus on expanding the theoretical method of dynamic modeling and analysis for harmonic gears, in order to improve the theoretical model’s precision. Moreover, the numerical method of wear analysis for harmonic gears remains to be studied further, especially the wear analysis of space mechanism under extreme service environment.

## Figures and Tables

**Table 1:** Adopted parameter values.

Parameter	Symbol	Flexspline	Circular Spline
Tooth number	z	200	202
Modul/mm	m	0.3	0.3
Pressure angle/°	α	20	20
Tooth height coefficient	$h_{a}^{*}$	1	1
Bottom clearance	$c^{*}$	0.35	0.35
Tooth width/mm	b	12	12
Modification coefficient	x_i_	3.32	3.32
Radial clearance/mm	δ	0.036	0.036
Materials density/kg·m^−3^	ρ	7750	7810
Young’s modulus/G·pa	E	204	200
Poisson’s coefficient	ν	0.3	0.277
Hardness, H/HBS	H	200	200
Load torque/N·m	T	16	16

## Data Availability

Not applicable.

## References

[1] Raviola A., De Martin A., Guida R., Jacazio G., Mauro S., Sorli M. Harmonic drive gear failures in industrial robots applications: An overview. Proceedings of the 6th European Conference of the Prognostics and Health Management Society.

[2] Schafer I., Bourlier P., Hantschack F., Roberts E.W., John C. Space lubrication and performance of harmonic drive gears. Proceedings of the 11th Esmats Symposium.

[3] Gill S., Forster D.J., Rowntree R.A. Thermal vacuum performance of cycloid and harmonic gearboxes with solid (MoS2) and liquid (Braycote601) lubrication. Proceedings of the 5th European Space Mechanisms and Tribology Symposium.

[4] Heimerdinger H., Schmid M. Life test experience on a harmonic-drive-based actuator. Proceedings of the 6th European Space Mechanisms and Tribology Symposium.

[5] Maniwa K., Obara S. (2007). Lubrication mechanism between wave generator and flexspline in strain wave gearing under in vacuum and air environment. J. Jpn. Soc. Tribolog..

[6] Maniwa K., Obara S. (2007). Mixed lubrication analysis between wave generator and flexspline in strain wave gearing. J. Jpn. Soc. Tribolog..

[7] Li J.Y. (2012). Failure Mechanism Theory and Accelerated Life Testing Method Research for Space Lubrication Harmonic Drive. Ph.D. Thesis.

[8] Li J.Y., Wang J.X. (2016). Accelerated life model for harmonic drive under adhesive wear. J. Tribol..

[9] Raviola A., De Martin A., Sorli M. (2022). A Preliminary Experimental Study on the Effects of Wear on the Torsional Stiffness of Strain Wave Gears. Actuators.

[10] Hugnell A.B.J., Bjoerklund S., Andersson S. (1996). Simulation of mild wear in a cam-follower contact with follower rotation. Wear.

[11] Ivsshov E.N., Nekrasov M.I. (1984). Calculation of the Wear of Harmonic Gears. Sov. Eng. Res..

[12] Bo S.X. (2008). Analysis of Flexspline Stress and Tooth Surface Wear in Harmonic Gear Drive. Master’s Thesis.

[13] Jia H., Xin H.B. (2022). Study on Lubrication Characteristics of Novel Forced Wave Generator of Harmonic Drive without Flexible Bearing. Materials.

[14] Andersson S. (1975). Partial EHD Theory and Initial Wear of Gears.

[15] Flodin A., Andersson S. (2000). Simulation of mild wear in helical gears. Wear.

[16] Flodin A., Anderson S. (2001). A simplified model for wear prediction in helical gears. Wear.

[17] Karpat F., Ekwaro-Osire S. (2008). Influence of tip relief modification on the wear of spur gears with asymmetric teeth. Tribol. Trans..

[18] Tunalioğlu M.Ş., Tuç B. (2014). Theoretical and experimental investigation of wear in internal gears. Wear.

[19] Shen Z., Qiao B., Yang L., Luo W., Yang Z., Chen X. (2021). Fault mechanism and dynamic modeling of planetary gear with gear wear. Mech. Mach. Theory.

[20] Shen Z., Qiao B., Yang L., Luo W., Chen X. (2019). Evaluating the influence of tooth surface wear on TVMS of planetary gear set. Mech. Mach. Theory.

[21] Huang D., Wang Z., Kubo A. (2020). Hypoid gear integrated wear model and experimental verification design and test. Int. J. Mech. Sci..

[22] Chen Z., Shao Y. (2013). Mesh stiffness calculation of a spur gear pair with tooth profile modification and tooth root crack. Mech. Mach. Theory.

[23] Zhang J., Bian S.Y., Lu Q. (2017). Quasi-static-model-based wear analysis of spur gears. J. Mech. Eng..

[24] Zhou C., Lei Y., Wang H.B. (2018). Adhesive wear models for helical gears under quasi-static and dynamic loads. J. Mech. Eng..

[25] Zhou C., Wang H. (2018). An adhesive wear prediction method for double helical gears based on enhanced coordinate transformation and generalized sliding distance model. Mech. Mach. Theory.

[26] Chen Z.G., Shao Y.M. (2015). Dynamic features of planetary gear train with tooth errors. Proc. Inst. Mech. Eng. Part C J. Mech. Eng. Sci..

[27] Ma H., Zeng J., Feng R.J. (2016). An improved analytical method for mesh stiffness calculation of spur gears with tip relief. Mech. Mach. Theory.

[28] Sun Y.N., Ma H., Hungfu Y.F. (2018). A revised time-varying mesh stiffness model of spur gear pairs with tooth modifications. Mech. Mach. Theory.

[29] Li J.Y., Wang J.X., Zhou G.W., Pu W.E.I., Wang Z.H. (2015). Accelerated life testing of harmonic driver in space lubrication. Proc. Inst. Mech.Eng. Part J J. Eng.Tribol..

[30] Luo Y., Baddour N., Liang M. (2019). Dynamical modeling and experimental validation for tooth pitting and spalling in spur gears. Mech. Syst. Signal Process..

[31] Xiao Z., Shi X., Wang X., Ma X., Han Y. (2021). Lubrication analysis and wear mechanism of heavily loaded herringbone gears with profile modifications in full film and mixed lubrication point contacts. Wear.

[32] Wang H., Zhou C., Lei Y., Liu Z. (2019). An adhesive wear model for helical gears in line-contact mixed elastohydrodynamic lubrication. Wear.

[33] Archard J.F., Hirst W. (1956). The wear of metals under unlubricated conditions. Proc. R. Soc. Lond. Ser. A.

[34] Archard J.F. (1953). Contact and rubbing of flat surfaces. J. Appl. Phys..

[35] Li J.Y., Wang J.X., Zhou G.W. (2013). Failure mechanism of harmonic drivers for space. J. Tribol..

[36] Masjedi M., Khonsari M.M. (2014). Theoretical and experimental investigation of traction coefficient in line-contact EHL of rough surfaces. Tribol. Int..

[37] Masjedi M., Khonsari M. (2015). An engineering approach for rapid evaluation of traction coefficient and wear in mixed EHL. Tribol. Int..

[38] Kayabashi O., Erzincanli F. (2007). Shape optimization of tooth profile of a flexspline for a harmonic drive by finite element modelling. Mater. Des..

[39] Janakiraman V. (2013). An Investigation of the Impact of Contact Parameters on the Wear Coefficient. Ph.D. Thesis.

[40] Yang C., Hu Q., Liu Z., Zhao Y., Cheng Q., Zhang C. (2020). Analysis of the partial axial load of a very thin-walled spur-gear (flexspline) of a harmonic drive. Int. J. Precis. Eng. Manuf..

